# Is it possible to define reference values for radiographic parameters evaluating juvenile flatfoot deformity? A case-control study

**DOI:** 10.1186/s12891-020-03854-6

**Published:** 2020-12-11

**Authors:** Johannes Hamel, Hubert Hörterer, Norbert Harrasser

**Affiliations:** 1 Schoen Clinic Munich-Harlaching, Specialist Centre for Foot and Ankle Joint Surgery, Harlachinger Str. 51, 81547 Munich, Germany; 2 Klinik für Allgemeine, Unfall- und Wiederherstellungschirurgie, Klinikum der Universität München, LMU München, Nussbaumstrasse 20, 80336 Munich, Germany; 3 Department of Orthopedics and Sports Orthopedics, Technical University Munich, Klinikum rechts der Isar, Ismaninger Str. 22, 81675 Munich, Germany

**Keywords:** Pes planus, Flatfeet - Planovalgus deformity, Radiographic measurements, Talometatarsal index

## Abstract

**Background:**

Numerous radiographic parameters are described to evaluate juvenile flexible flatfeet. Reference values for these measurements are based on few studies. The purpose of this study was to determine boundary values among the most widely used radiographic measurements to evaluate juvenile flatfeet.

**Methods:**

Twenty-two patients with normal hind-, midfoot configuration (group A: control group; 22 ft, mean age: 12,1 years) and 19 patients with flatfoot deformity (group B: study group; 22 ft, mean age: 12,4 years) were retrospectively analyzed. Nine radiographic parameters were measured (Talocalcaneal-angles, Calcaneal-pitch-angle, Costa-Bartani-angle, Talo-metatarsal-I-angles, Talo-first-metatarsal-base-angle, Talo-navicular-coverage, Calcaneus-fifth-metatarsal-angle). ROC curve analysis was used to calculate optimal differentiating thresholds of each parameter.

**Results:**

Four out of nine parameters (TC-dp, TC-lat, Calc-MTV, Calc-P) were not statistically different between the groups and their ability to distinct between normal foot and flatfoot was low (AUC values = 0,660 - 0,819). Calculation of reference values for these parameters was not performed due to threshold ranges between the groups of > 10°. Reference values could be defined only for three parameters: TMTInd >(−)31°, TMTIB >(−)7,5°, TMT-lat > (−)13,5°. The TMTInd was shown to be a very reliable and valid combination of two measurements (TMTIB and TMT-lat) in the differentiation of normal feet and flatfeet (AUC = 0,998).

**Conclusion:**

The calculation of reference values for established radiographic parameters used to evaluate juvenile flatfeet is difficult for most parameters. The TMTInd as a combination of TMTIB and TMT-lat has been shown to be reliable and valuable to distinct normal feet from flatfeet.

## Background

The foot of healthy, typically developing children is flexible and flat, and therefore different from the adult population [1, 2]. The symptomatic flexible juvenile flatfoot is of particular importance to orthopedics and foot surgeons due to its high occurrence and variable treatment strategies [3]. On the basis of patients’ history, clinical investigation and X-ray findings a decision is usually made regarding conservative or operative treatment. Radiography of the foot in two planes under full weight bearing conditions is of crucial importance due to the possibility to objectively quantify the deformity. For this reason, several radiographic parameters have been described [4]. Nevertheless, most of the data on reference values for these measurements to differentiate between flatfoot and “normal” foot are based on experience, studies on adult flatfeet or studies with high probability of bias [5–9]. Normal values of radiographic parameters describing the foot shape of healthy children and adolescents in the flatfoot-relevant age from 10 to 14 years are only based on few studies (Table 1).

**Table 1:** Radiographic parameters investigated in the current study with so far published reference values in children/adolescents

Author	Dorsoplantar					Lateral			
	TC-dp	TNC	TMTIB	Calc-MTV	TMTI-dp	Calc-P	Costa-B	TMTI-lat	TC-lat
**Davids [7]**	-	20° ± 9.8°	-	-	10° ± 7°	17° ± 6°	-	13° ±7.5° (1–35°)	49° ± 6.9°
**Vanderwilde [8]**	-	-	-	-	6° ± 7°	-	-	8° ±6°	39° ± 7°
**DePellegrin [9]**	-	-	-	-	-	20–30°	120–125°	-	15–20°
**Hell[3]**	Newborn: 40° (25–55°)	20° ± 9.8°	-	0°	10° ± 7°	-	120–125°	newborn: 20° (9–31°) 8 years of age: 5° (10–18°)	40° (25–55°)
**Bourdet [6]**	35–40°	-	-	5–30°	-	15–20°	-	0–10°	-
**Benedetti [4]**	inconsistent	0° ± 4°	-	-	-	-	120–125°	0° ± 4°	inconsistent

To the best of our knowledge no comparative studies exist who attempted to define reference values for radiological parameters most often used in clinical praxis to evaluate juvenile flexible flatfoot deformity. Given the lack of information about cut-off values for these measurements, we performed this study to provide data for better interpretation and quantification of flatfoot radiographs and to analyze the reliability and validity of specific measurements.

## Methods

This retrospective study was approved by the institutional review board at our hospital. A total of 44 ft (41 patients) were included in the analysis. *Group 1* was referred to as the “normal foot group” (22 ft from 19 patients; average age: 12.1 years (range: 10.2–14.8 years)). For ethical reasons, it is not appropriate to perform X-rays on healthy children’s feet for study purposes. Thus, we included patients in this group who had visited our outpatient department because of minor forefoot misalignments or deformities and had therefore undergone standard X-rays of the foot in two planes under full weight-bearing. The diagnoses for these patients were: 4x polydactyly, 4x hallux valgus, 2x dorsal bumps at the tarsometatarsal region, 1x M. Koehler II, 5x unclear pain, 3x calcaneal apophysitis, 3x exclusion of flatfoot. In this group no clinically recognizable tarsal deformities were present. Further exclusion criteria were: Previous operations or fractures of the foot, history of trauma of the foot within 4 weeks or presence of any tarsal coalitions. It can be assumed that this cohort consisted of individuals who can be considered “normal” in terms of tarsal architecture of the foot. *Group 2* was referred to as the “flatfoot group” (22 ft from 22 patients; average age: 12.4 years (range: 10.7–14.2 years)) and consisted of consecutive patients who had undergone a surgical procedure (arthroereisis and/or combination of osteotomies) by the first author in 2019 due to a flexible juvenile flatfoot deformity. The indication for surgery was made in accordance with other authors if anamnestic/clinical findings (e.g. pain, low endurance when standing and walking, shortening of gastrocnemius, excessive callosities in the area of ​​the medial foot arch) and radiological parameters (at least two pathologic values of radiographic measurements cited in this work) were suggestive for symptomatic flatfoot deformity and conservative treatment had failed over 6 months [9–17].

The evaluation of X-rays (foot in dorso-plantar and lateral view, standing with full weight bearing) was carried out digitally using the DICOM system. The measurements of Group 2 were performed on preoperative images. All parameters were measured by two authors of the study (foot and ankle surgeons) on two separate occasions with the order of images randomized. Each observer made measurements independently and was blinded to both patient identification and the others’ results.

**Fig. 1:**
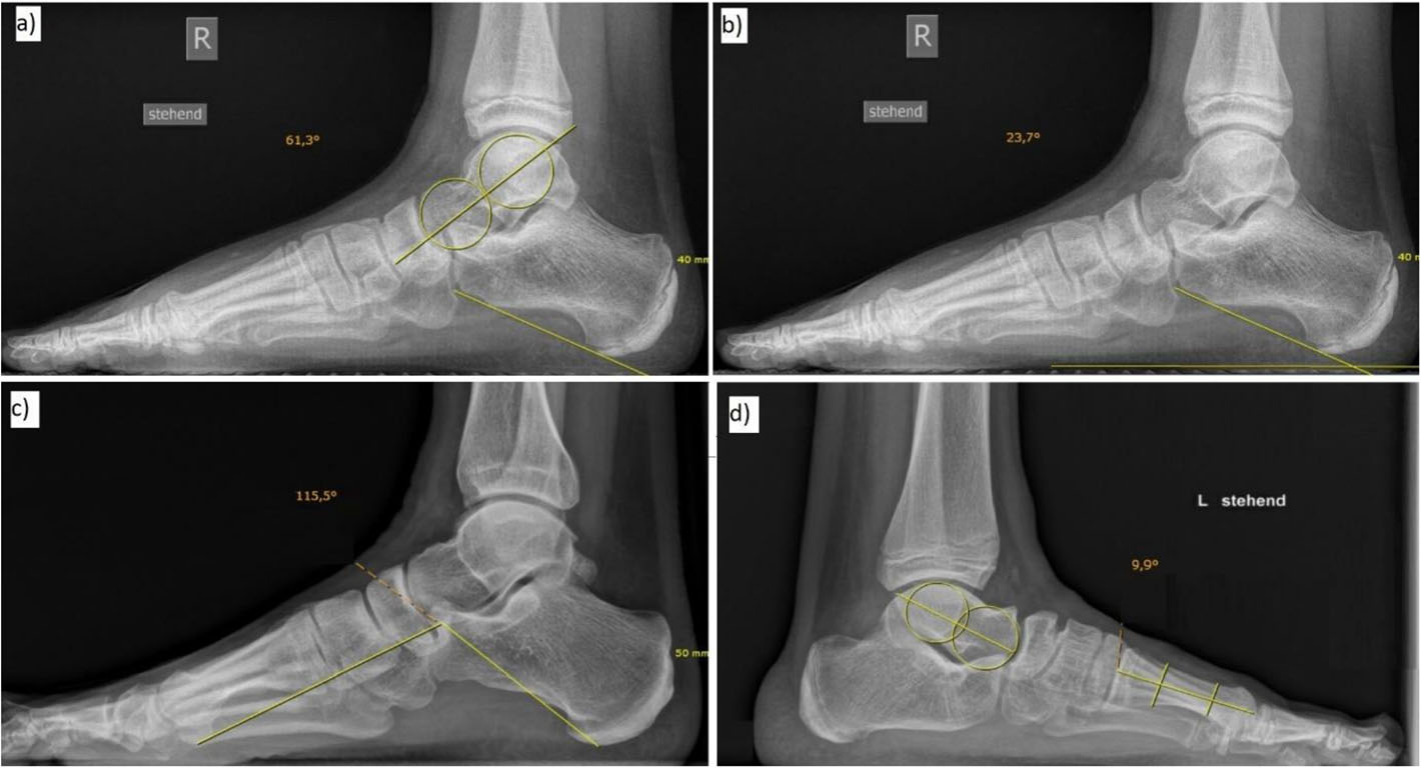
Radiographic parameters evaluated in the lateral view: **a** Talocalcaneal angle (TC-lat), (**b**) calcaneal-pitch angle (Calc-P), (**c**) Costa-Bartani angle (Costa-B), (**d**) Talo-first-metatarsal angle (TMTI-lat)

**Fig. 2:**
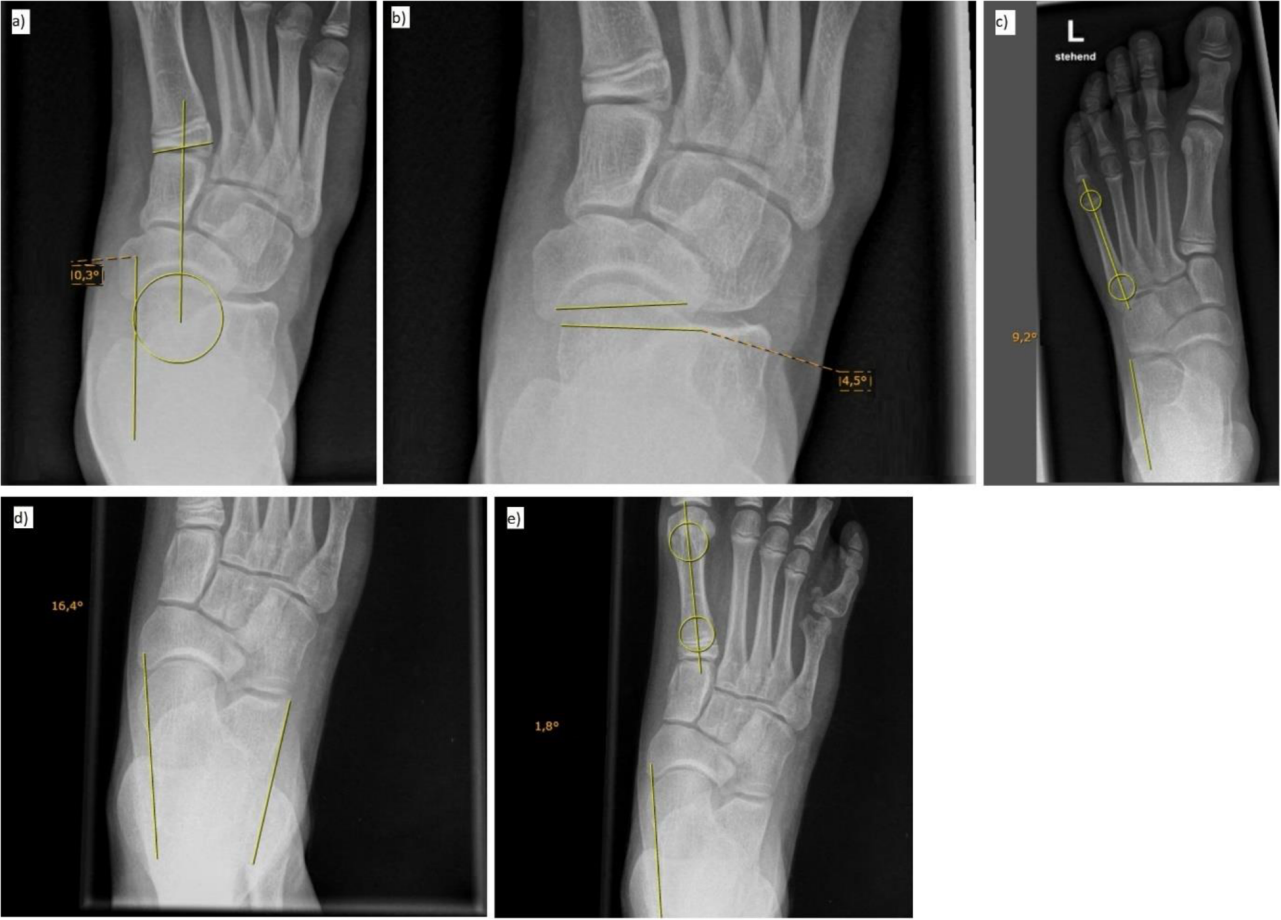
Radiographic parameters evaluated in the dorsoplantar view: **a** Talo-first-metatarsal-base angle (TMTIB), (**b**) Talonavicular coverage (TNC), (**c**) Calcaneus-fifth-metatarsal angle (Calc-MTV), (**d**) Talocalcaneal angle (TC-dp), (**e**) Talo-first-metatarsal angle (TMTI-dp)

The following X-ray parameters were measured (Figs. 1 and 2; “minus sign” if distal line in relation to proximal line of the angle directs towards abduction in the d.-p. view or elevation in the lateral view):

• Lateral view: Talocalcaneal angle (TC-lat), calcaneal pitch angle (Calc-P), Costa-Bartani angle (Costa-B), talo-first-metatarsal angle (TMTI-lat).

• Dorsoplantar view: Talo-first-metatarsal-base angle (TMTIB), talonavicular coverage (TNC), calcaneus-fifth-metatarsal angle (Calc-MTV), talocalcaneal angle (TC-dp), talo-first-metatarsal angle (TMTI-dp).

In addition, a combination of parameters in both planes were analyzed as in the authors’ experience these values analyze flatfeet more accurately than do single measurements in one plane (talometatarsal index (TMTInd [18, 19]): sum of TMTIB and TMTI-lat; talocalcaneal index (TCInd): sum of TC-lat and TC-dp).

The measurement of these radiographic angles is based on characteristic lines sometimes defined in a vague manner in the current literature [4–6, 8, 9, 20, 21]. In the lateral view, the following definitions were applied:

• Longitudinal axis of the talus: Line connecting the centers of two incircles in the area of ​​the talar body and talar head.

• Longitudinal axis of the calcaneus: Line touching tangentially the most plantar areas of the anterior and posterior part of the calcaneus.

• Longitudinal axis of the first metatarsal: Line bisecting the proximal and distal shaft diameter at the meta-diaphyseal junction.

• Costa-Bartani angle: Angle between the connecting line of the most plantar point of the talar head contour/the most plantar point of the calcaneus and the connecting line of the most plantar point of the medial sesamoid bone of the great toe/the most plantar point of the talar head contour.

In the dorsoplantar view, the following definitions were applied:

• Longitudinal axis of the talus: Tangent line parallel to the medial contour of the talus.

• Longitudinal axis of the calcaneus: Tangent line parallel to the lateral contour of the calcaneus (a prominent peroneal tubercle was not considered).

• Metatarsal axes of the first and fifth ray: Connecting line of the centers from two incircles at the levels of the proximal and distal meta-diaphyseal junction.

• Talo-first-metatarsal-base angle (TMTIB): Angle between the longitudinal axis of the talus and a line through the geometric center of the talar head (determined by the incircle of the talar head) and the center of the first metatarsal base.

• Talonavicular coverage (TNC): Points are marked on the most medial and lateral margins of the articular surface of the talus, and a line is drawn connecting these two points. Similar points are marked on the most medial and lateral margins of the articular surface on the navicular, and a line is drawn connecting these two points. The angle between these two lines was defined as TNC.

*Statistical analyses* were conducted with use of GraphPad Prism8 (GraphPad Software, San Diego, CA 92108). For all parameters, descriptive statistics were calculated. Quantitative parameters are described by their mean and standard deviation (SD). Differences between the means and all other parameters in both groups were determined using analysis of variance (ANOVA). A Bonferroni correction was used as a post-hoc test (*p* < 0.05: significant; *p* < 0.01: highly significant). To evaluate correlation between 2 quantitative parameters, Pearson or Spearman coefficients were computed. Corresponding ROC curves (receiver operating characteristic) were created as a method for evaluating the X-ray parameters with regard to their ability as an analysis strategy and the area under the ROC curves (AUC: Area under the curve) was calculated accordingly [22]. It was assumed that a threshold range of ≤10° would be acceptable to define a true reference value. In this area of overlap normal feet and flatfeet would not be distinguishable. The interobserver reliability was determined using Pearson correlation analysis, the intraobserver reliability was assessed with ICC (Intraclass Correlation Coefficient). Inter- and intraobserver reliability was classified as minimal (correlation coefficient (CC) ≤ 0.25), low (0.26 < CC < 0.5), moderate (0.5 ≤ CC < 0.7), high (0.7 ≤ CC < 0.9), and excellent (CC ≥ 0.9) [23].

## Results

The results of radiological measurements of both groups with corresponding *p*-values are shown in Table 2. All parameters but the talocalcaneal angle (dorsoplantar-view), calcaneal pitch angle, calcaneus-fifth-metatarsal angle, talocalcaneal (lateral-view) were statistically different between the groups, and thus able to differentiate between normal feet and flatfeet. Reliability testing (Table 3) of nearly all parameters, except for talocalcaneal angle (dorsoplantar-view), revealed high to excellent reproducibility of measurements.

**Table 2:** Values of radiographic parameters of both groups with corresponding *p-*values. SD: standard deviation. n.s.: not significant. Note: “minus sign” of values if distal line in relation to proximal line of the angle directs towards abduction in the d.-p. view or elevation in the lateral view

View	Parameter	Mean (±SD)		***p***-value
		Group 1	Group 2	
**d.-p.**	Talocalcaneal angle	21.6(±4.5)	22.6 (±7.2)	n.s.
	Talonavicular coverage	−17.1(±7.5)	−34.1(±8.3)	< 0.01
	Talo-first-metatarsal-base angle	−10.0(±4.9)	−21.3(±6.9)	< 0.01
	Calcaneus-fifth-metatarsal angle	−5.2(±4.9)	−12.0 (±4.9)	n.s.
	Talo-first-metatarsal angle	−5.0 (±5.1)	−13.9(±7.7)	< 0.01
**Lateral**	Calcaneal-pitch angle	19.0(±5.8)	12.2(±5.3)	n.s.
	Costa-Bartani angle	125.5(±5.9)	141.4(±7.2)	< 0.01
	Talo-first-metatarsal angle	−12.1(±5.7)	−30.5 (±9.3)	< 0.01
	Talocalcaneal angle	53.5(±7.5)	60.1 (±6.3)	n.s.
**d.-p. and lateral**	Talometatarsal index	−22.1(±8.4)	−58.1(±13.7)	< 0.01
	Talocalcaneal index	75.1(±9.0)	82.7(±7.5)	n.s.

**Fig. 3:**
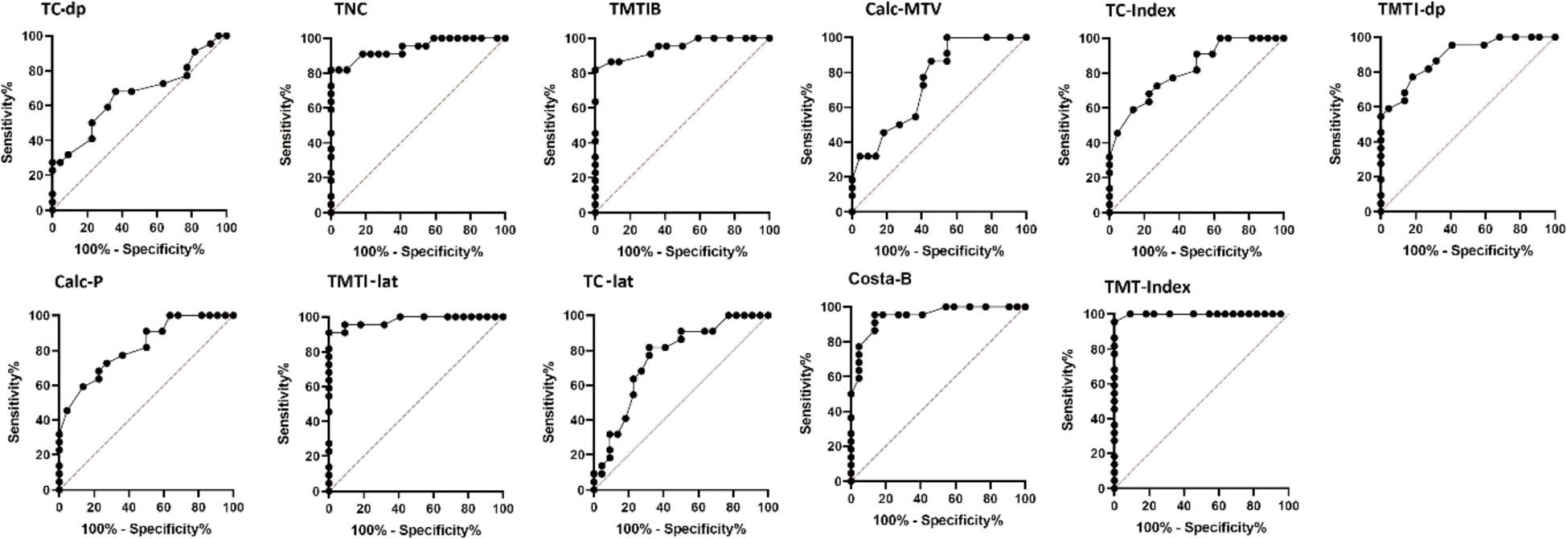
ROC curves of different radiographic parameters; TC-lat: Talocalcaneal angle (lateral-view), Calc-P: Calcaneal-pitch angle, Costa-B: Costa-Bartani angle, TMTI-lat: Talo-first-metatarsal angle (lateral-view), TMTIB: Talo-first-metatarsal-base angle, TNC: Talonavicular coverage, Calc-MTV: Calcaneus-fifth-metatarsal angle, TC-dp: Talocalcaneal angle (dorsoplantar-view), TMTI-dp: Talo-first-metatarsal angle (dorsoplantar-view), TMTInd: Talometatarsal index, TCInd: Talocalcaneal index

**Fig. 4:**
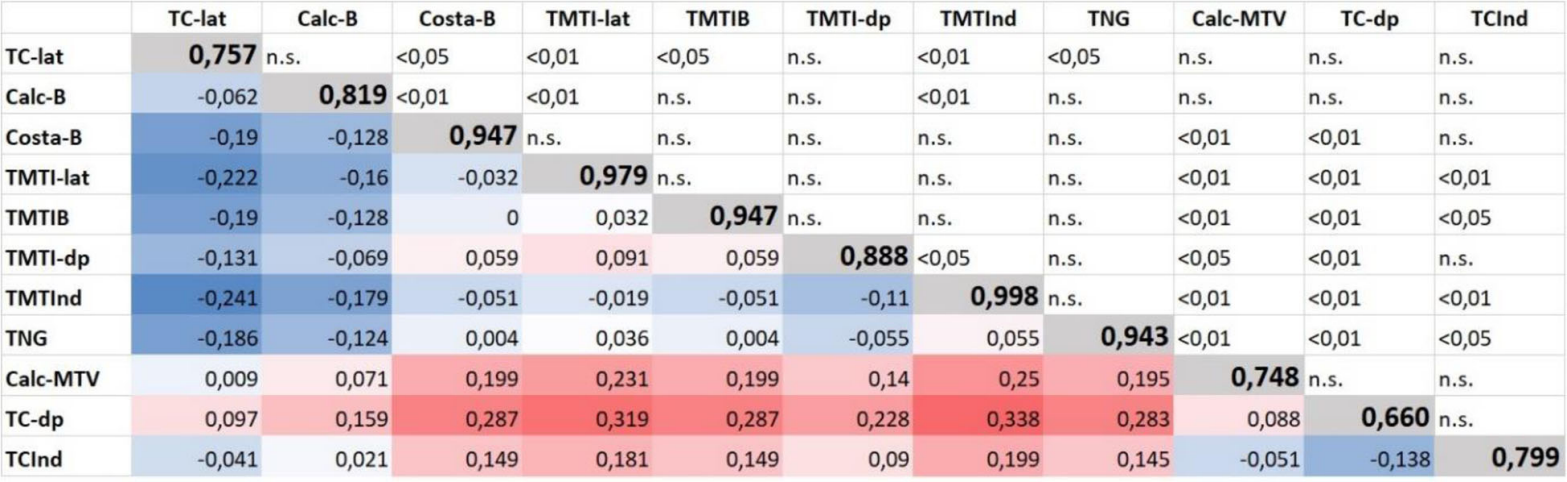
Representation of *p*-values of the AUC in comparison (upper triangular matrix; n.s.: not significant), the absolute AUC values (main diagonal, values marked in bold), and the absolute difference of the AUC values (color representation depending on the magnitude of absolute difference); Note: An AUC value of 1 corresponds to a 100% ability of the parameter to distinguish between normal foot and flatfoot, an AUC value of 0.5 indicates a 50% probability. Representation after DeLong [12]. TC-lat: Talocalcaneal angle (lateral-view), Calc-P: Calcaneal-pitch angle, Costa-B: Costa-Bartani angle, TMTI-lat: Talo-first-metatarsal angle (lateral-view), TMTIB: Talo-first-metatarsal-base angle, TNC: Talonavicular coverage, Calc-MTV: Calcaneus-fifth-metatarsal angle, TC-dp: Talocalcaneal angle (dorsoplantar-view), TMTI-dp: Talo-first-metatarsal angle (dorsoplantar-view), TMTInd: Talometatarsal index, TCInd: Talocalcaneal index

ROC curves are given in Fig. 3, descriptive values (including AUC values) are shown in Fig. 4. The talometatarsal index shows the highest AUC value compared to all other parameters. Further ROC analysis regarding the ability of the different parameters to distinct between both groups, i.e. to define a true reference value, is given in Table 4. Within this calculated threshold range of every single radiographic parameter the transition between normal foot and flatfoot can be assumed. A threshold range as low as 3 ° was calculated for the talometatarsal index (talo-fist-metatrsal-base angle = 10°, talo-first-metatarsal angle (lateral-view) = 6,5°), all others ranged from 11° to 22°.

**Table 3:** Intra−/interobserver reliability of radiographic parameters

Parameter	Intraobserver reliability	Interobserver reliability
	Mean of Author A and B	Author A vs. B
Talocalcaneal angle (dorsoplantar-view)	0.77	0.65
Talonavicular coverage	0.92	0.92
Talo-first-metatarsal-base angle	0.90	0.90
Calcaneus-fifth-metatarsal angle	0.92	0.92
Talo-first-metatarsal angle (dorsoplantar-view)	0.90	0.90
Calcaneal-pitch angle	0.98	0.94
Costa-Bartani angle	0.96	0.90
Talo-first-metatarsal angle (lateral-view)	0.94	0.95
Talocalcaneal angle (lateral-view)	0.96	0.96
Talometatarsal index	0.91	0.90
Talocalcaneal index	0.89	0.90

**Table 4:** ROC analysis: Representation of different radiographic parameters and their ability to proof (100% sensitivity, 2nd column) or exclude (100% specificity, 3rd column) the presence of a normal foot at given upper or lower boundary values. The “threshold range” (4th column) describes the area of overlap of every parameter, where no clear definition of whether the measurement describes a normal foot or a flatfoot can be made. In the 5th and 6th column the number of feet that could be clearly classified as normal foot or flatfoot according to the upper and lower limits of the threshold ranges are shown

Parameter	Threshold value [°] for proof/exclusion of normal feet		Threshold range [°]	Number of feet classifiable according to threshold value	
	100% Sensitivity	100% Specificity		Normal feet (***n*** = 22)	Flatfeet (***n =*** 22)
Talocalcaneal angle (dorsoplantar-view)	< 12.5	> 26.5	14	1	6
Talonavicular coverage	> − 16	<− 28.5	12,5	9	18
Talo-first-metatarsal-base angle	> − 7.5	<−17.5	10	9	18
Calcaneus-fifth-metatarsal angle	> − 3.5	<−16.5	13	9	4
Talo-first-metatarsal angle (dorsoplantar-view)	> − 1.5	<−12.5	11	7	12
Calcaneal-pitch angle	> 21.5	< 10.5	11	9	7
Costa-Bartani angle	< 124.5	> 141.5	17	10	11
Talo-first-metatarsal angle (lateral-view)	> − 13.5	<−20	6,5	12	20
Talocalcaneal angle (lateral-view)	< 47.5	> 69.5	22	2	2
Talometatarsal index	> − 31	<−34	3	20	21
Talocalcaneal index	< 66	> 88	22	7	5

## Discussion

The juvenile flexible flatfoot deformity is pathomorphologically characterized by an increased eversion of the peritalar complex and a destabilization of the first ray [12]. For quantification of the deformity various radiological parameters are described [4–6, 8, 20, 24]. Surprisingly, the available evidence of certain thresholds or values is sparse. In the present study, we were able to calculate threshold ranges of established radiographic parameters based on data from surgically treated symptomatic juvenile flatfeet and normal feet. Four out of nine parameters (talocalcaneal angle (dorsoplantar-view), talocalcaneal angle (lateral-view), calcaneus-fifth-metatarsal angle, calcaneal pitch angle) were not statistically different between the groups and their ability to distinct between normal foot and flatfoot (AUC values, Fig. 4) was shown to be low. Moreover, definition of reference values for these measurements is inaccurate. Additionally, we found the talometatarsal index as a very reliable and valid combination of two measurements with a very small threshold range between − 31 to − 34°.

Additionally, the given values often differ considerably and the studies are highly subjected to some sort of bias. For example, Vanderwilde et al. mainly reports on younger children and only included nine children ≥9 years. In addition, the dorsoplantar X-ray image which is exemplary shown in the original paper appears to be taken not with the foot under full weight bearing but rather “knee flexed” [8]. Another study on this topic was published by Davids et al., whereby the unaffected side of patients with hemiparesis served as a control [7]. The dorsoplantar pictures shown in the paper do not allow for visualization of the hindfoot to define the longitudinal axes of the calcaneus or talus, making these measurements probably unreliable. Additionally, the setting of normal values ​​is reported insufficiently due to the fact that children included in the study were between 5 to 17 years (on average 10 years). In this period of life, the foot undergoes relevant physiologic changes of shape and a normal value calculated is possibly affected by some sort of systematic error. Nevertheless, for talo-first-metatarsal angle (lateral-view), talocalcaneal angle (lateral-view) and talonaviculare coverage there is high congruency with our values with only talo-first-metatarsal angle (dorsoplantar-view) showing relevant difference from our measurements.

Five out of nine parameters were able to significantly differentiate normal from flat feet, whereas, the following four parameters missed: talocalcaneal angle (lateral-view), talocalcaneal angle (dorsoplantar-view), calcaneal pitch angle and calcaneus-fifth-metatarsal-base angle. It must be concluded that in the current study these four parameters failed as valid measurements to diagnose juvenile flatfoot deformity. A radiological parameter classifying the severity of a flatfoot deformity is suitable to the extent that it can be measured reliably on the one hand (see Table 2) and has a good selectivity between normal and pathologic feet on the other hand. In this context, Table 3 shows the parameters’ selectivity of the tested angles and it can be concluded: The lower the threshold range, the more accurate a reference value can be defined. Then again, if a parameter has a large threshold range, a large overlap area of measurements indicating normal foot or flatfoot must be expected. We believe, a parameter with a threshold range > 10° must be interpreted with conscious. In this context, only the talometatarsal index and its sub-parameters talo-first-metatarsal-base angle and talo-first-metatarsal angle (lateral-view) had acceptable values with ranges ≤10°. The following reference values for normal feet of adolescents between 10 and 14 years could therefore be defined: Talometatarsal index >(−)31°, talo-first-metatarsal-base angle >(−)7.5°, talo-first-metatarsal angle (lateral-view) > (−)13.5°.

### Analysis of individual parameters

#### Talocalcaneal angles

The reliability testing for the talocalcaneal angle (dorsoplantar-view) was 0.77 and 0.65, and therefore significantly lower in comparison to all other parameters. The main difficulty in the measurement of this angle is the fact, that the shape of the talus and calcaneus at the level of their bodies is not always easily definable. The talocalcaneal angle (dorsoplantar-view) shows a comparatively low ability to distinct between the two groups (AUC = 0,660). This is higher for the talocalcaneal angle (lateral-view) (AUC = 0,757). In the end it must be noted, that talocalcaneal angles could hardly distinguish between the groups, and therefore it was not possible to set a reference value. The sum of both talocalcaneal angles led to a slightly improved performance to distinct between the groups (talocalcaneal index with AUC = 0,799), but nonetheless, the usefulness of the parameters is rather low. This also corresponds to the experience of other authors who used the same parameter with idiopathic clubfoot decades ago [25].

#### Costa-Bartani angle

The Costa-Bartani angle shows a particularly high reliability. In contrast, the distinction between the groups was only moderate with only half of the feet being correctly allocated to either groups. This can be explained by the large threshold range of 17 °. Therefore, the definition of a reference value is not reasonable. The Costa-B angle correlates poorly with the angular dimensions that characterize the talonavicular alignment in the transverse plane (Pearson-correlation to talonaviculare coverage = − 0.30, to talo-first-metatarsal-base angle = − 0.34), but better with those parameters reflecting the alignment in the sagittal plane (Pearson-correlation to talo-first-metatarsal angle (lateral-view) = − 0.63). One major drawback of the parameter in our experience is the difficulty to clearly determine the distal point on the sesamoid, at least in childhood.

#### Talometatarsal angles

All talometatarsal angles showed good intra- and interobserver reliability, some of which is well above 0.9. Establishing the longitudinal axis of the talus in the sagittal plane with the help of two incircles has proven effective. In the transversal plane it is noticeable, that the talo-first-metatarsal angle (dorsoplantar-view) and the talo-first-metatarsal-base angle differ from one another in the two groups, but this is not statistically significant. It is therefore important whether one uses the talo-first-metatarsal-base angle (which measures the conditions at the talonavicular joint more specifically) or the talo-first-metatarsal angle (dorsoplantar-view) (which is more susceptible to concomitant forefoot deformities, e.g. Metatarsus primus varus, skew-foot) to calculate the talometatarsal index.

The talometatarsal index showed the highest discrimination rate (AUC = 0.998) of all parameters. It was also possible to define a reference value between the two groups on an overlap area of only 3 °. 20 of 22 ft of the normal group and 21 of 22 flatfeet could be assigned to the appropriate group by the talometatarsal index alone. Due to the small threshold range a definition of reference values was also possible for the talo-first-metatarsal-base angle and the talo-first-metatarsal angle (lateral-view).

In the flatfoot group, the percentual fraction of the talo-first-metatarsal-base angle as part of the talometatarsal index was 41% on average and that of the talo-first-metatarsal angle (lateral-view) 59%, respectively. It is noteworthy, however, that the proportion of the talo-first-metatarsal-base angle ranges from 22 to 61%. This shows, that there is a wide range of individual fluctuations with regard to the involvement of the sagittal plane and transverse plane in the flatfoot deformity. It is also a confirmation of the very different planar dominance of the talocalcaneo-navicular complex in every single flatfoot [6]. This observation can also be used to conclude, that both planes must be taken into account (as ensured by the talometatarsal index) to evaluate the flatfoot deformity correctly and in its entire form.

#### Talonavicular coverage

Measuring talonaviculare coverage showed a very high reliability, although it is sometimes not easy to define the boundaries of the articular surface, especially at the level of the talar head. In addition, talonaviculare coverage showed approximately the same AUC value (0.943) as compared to the talo-first-metatarsal-base angle (0.947). However, a reference value was not easily definable with a threshold range of 12.5°.

#### Calcaneal pitch angle, calcaneus-fifth-metatarsal angle

In contrast to all other parameters, these two radiographically well reproducible angles do not allow any statement about the positional relationship between the talus and the subtalar footplate. For basic considerations, these parameters alone are less suitable to characterize the flatfoot deformity. Additionally, the threshold ranges are particularly high and AUC values are far under 0.9. Definition of reference values for these parameters was not helpful.

A limitation of the present study may be the fact, that in the control group all patients had some kind of foot disorders and the term “normal” must be interpreted with conscious. Nevertheless, none of the patients had pathologies influencing the foot arch or causing flatfoot-like deformities. Additionally, all patients in this group had clinically a normal foot shape in the area of interest, namely the tarsal region. We are well aware, that classifying a flatfoot is sometimes subjective, especially if the deformity is not too pronounced. This could influence the measurements conducted in the study group and making the borders between the groups blur. On the other hand, surgery was only performed on patients with relevant pathology (clinically and radiographically), and therefore it can be assumed, that our study group consisted only of clear diagnoses regarding a flatfoot deformity. Another weakness of the current study is the small sample size. Even though, the most relevant studies with similar questions do have very similar patient numbers, studies with higher numbers are needed to confirm our findings [6–8].

## Conclusion

It was possible to define reference values for three parameters (talometatarsal index, talo-first-metatarsal-base angle, talo-first-metatarsal angle in the lateral-view) evaluating the juvenile flatfoot deformity. The talometatarsal index appears to be particularly suitable for the distinction between normal foot and juvenile flatfoot.

## Data Availability

The datasets used and/or analysed during the current study are available from the corresponding author on reasonable request.

## Abbreviations

TC-lat: Talocalcaneal angle
Calc-P: Calcaneal-pitch angle
Costa-B: Costa-Bartani angle
TMTI-lat: Talo-first-metatarsal angle
TMTIB: Talo-first-metatarsal-base angle
TNC: Talonavicular coverage
Calc-MTV: Calcaneus-fifth-metatarsal angle
TC-dp: Talocalcaneal angle
TMTI-dp: Talo-first-metatarsal angle
TMTInd: Talometatarsal index
TCInd: Talocalcaneal index
ICC: Intraclass Correlation Coefficient

## References

[1] Gijon-Nogueron G, Martinez-Nova A, Alfageme-Garcia P, Montes-Alguacil J, Evans AM (2019). International normative data for paediatric foot posture assessment: a cross-sectional investigation. BMJ Open.

[2] Uden H, Scharfbillig R, Causby R (2017). The typically developing paediatric foot: how flat should it be? A systematic review. J Foot Ankle Res.

[3] Hell AK, Doderlein L, Eberhardt O, Hosl M, von Kalle T, Mecher F, Simon A, Stinus H, Wilken B, Wirth T (2018). S2-guideline: pediatric flat foot. Z Orthop Unfall.

[4] Benedetti MG, Berti L, Straudi S, Ceccarelli F, Giannini S (2010). Clinicoradiographic assessment of flexible flatfoot in children. J Am Podiatr Med Assoc.

[5] Arunakul M, Amendola A, Gao Y, Goetz JE, Femino JE, Phisitkul P (2013). Tripod index: diagnostic accuracy in symptomatic flatfoot and cavovarus foot: part 2. Iowa Orthop J.

[6] Bourdet C, Seringe R, Adamsbaum C, Glorion C, Wicart P (2013). Flatfoot in children and adolescents. Analysis of imaging findings and therapeutic implications. Orthop Traumatol Surg Res.

[7] Davids JR, Gibson TW, Pugh LI (2005). Quantitative segmental analysis of weight-bearing radiographs of the foot and ankle for children: normal alignment. J Pediatr Orthop.

[8] Vanderwilde R, Staheli LT, Chew DE, Malagon V (1988). Measurements on radiographs of the foot in normal infants and children. J Bone Joint Surg Am.

[9] De Pellegrin M, Moharamzadeh D, Strobl WM, Biedermann R, Tschauner C, Wirth T (2014). Subtalar extra-articular screw arthroereisis (SESA) for the treatment of flexible flatfoot in children. J Child Orthop.

[10] Arbab D, Frank D, Bouillon B, Luring C, Wingenfeld C, Abbara-Czardybon M (2018). Subtalare screw Arthroereisis for the treatment of symptomatic, flexible Pes Planovalgus. Z Orthop Unfall.

[11] Cao L, Miao XD, Wu YP, Zhang XF, Zhang Q (2017). Therapeutic outcomes of Kalix II in treating juvenile flexible flatfoot. Orthop Surg.

[12] Fernandez De retana P, Alvarez F, Viladot R (2010). Subtalar arthroereisis in pediatric flatfoot reconstruction. Foot Ankle Clin.

[13] Martinelli N, Bianchi A, Martinkevich P, Sartorelli E, Romeo G, Bonifacini C, Malerba F (2018). Return to sport activities after subtalar arthroereisis for correction of pediatric flexible flatfoot. J Pediatr Orthop B.

[14] Memeo A, Verdoni F, Rossi L, Ferrari E, Panuccio E, Pedretti L (2019). Flexible juvenile flat foot surgical correction: a comparison between two techniques after ten Years' experience. J Foot Ankle Surg.

[15] Pavone V, Costarella L, Testa G, Conte G, Riccioli M, Sessa G (2013). Calcaneo-stop procedure in the treatment of the juvenile symptomatic flatfoot. J Foot Ankle Surg.

[16] Pavone V, Vescio A, Di Silvestri CA, Andreacchio A, Sessa G, Testa G (2018). Outcomes of the calcaneo-stop procedure for the treatment of juvenile flatfoot in young athletes. J Child Orthop.

[17] Roye DP, Raimondo RA (2000). Surgical treatment of the child's and adolescent's flexible flatfoot. Clin Podiatr Med Surg.

[18] Hamel J, Kinast C (2006). Der TMT-Index zur radiologischen Quantifizierung von Planovalgus-Deformitäten. FussSprungg.

[19] Richter M, Zech S (2013). Arthrorisis with calcaneostop screw in children corrects Talo-1st metatarsal-index (TMT-index). Foot Ankle Surg.

[20] Coughlin MJ, Kaz A (2009). Correlation of Harris mats, physical exam, pictures, and radiographic measurements in adult flatfoot deformity. Foot Ankle Int.

[21] Sensiba PR, Coffey MJ, Williams NE, Mariscalco M, Laughlin RT (2010). Inter- and intraobserver reliability in the radiographic evaluation of adult flatfoot deformity. Foot Ankle Int.

[22] DeLong ER, DeLong DM, Clarke-Pearson DL (1988). Comparing the areas under two or more correlated receiver operating characteristic curves: a nonparametric approach. Biometrics.

[23] Munro BH (2005). Statistical Methods for Health Care Research.

[24] Hamel J, Horterer H, Harrasser N. The talometatarsal-index ("TMT-Index"): A valuable X-ray parameter for differentiating between normal feet and planovalgus deformity in children and adolescents. Orthopade. 2020. 10.1007/s00132-020-03954-0. Online ahead of print. 10.1007/s00132-020-03954-0 32761421

[25] Beatson TR, Pearson JR (1966). A method of assessing correction in club feet. J Bone Joint Surg (Br).

